# 25-Year Comparison of Coronary Lesions Anatomy in Two Cohorts of French Canadians with Familial Hypercholesterolemia

**DOI:** 10.3390/jcm14020305

**Published:** 2025-01-07

**Authors:** Alex Lauziere, Diane Brisson, Gérald Tremblay, Sophie Bedard, Etienne Khoury, Daniel Gaudet

**Affiliations:** 1 Department of Medicine, Université de Montréal and ECOGENE-21, Chicoutimi, QC G7H 7K9, Canada; 2 Lipid Clinic, Chicoutimi Hospital, Chicoutimi, QC G7H 5H6, Canada

**Keywords:** familial hypercholesterolemia, coronary heart disease, prevention medicine

## Abstract

**Background:** Over the past decades, new treatments and guidelines have been introduced for the screening and management of familial hypercholesterolemia (FH). However, the impact of these medical and scientific advances on the characteristics and burden of coronary lesions over time in FH remains poorly documented. **Objective:** The primary goal of this study is to determine the characteristics of coronary lesions in HeFH patients who underwent coronary angiography within two distinct timeframes: the last five years versus those who had the procedure at the same hospital 25 years earlier. **Methods:** The characteristics of coronary lesions in 108 HeFH patients who consecutively underwent coronary angiography for CAD between 2017 and 2022 (2022 cohort) were compared to those of 240 patients who had an angiography between 1995 and 1998 (1998 cohort). **Results:** Compared to 1998, FH patients requiring coronary angiography were proportionally less numerous and significantly older in 2022 (*p* < 0.001). Although the number of coronary lesions (2.5 ± 1.5 in both cohorts) and the proportions of multi-vessel (70.4% vs. 70.2%), three-vessel (29.6% vs. 30.2%) and left main involvement (15.7% vs. 16.0%) did not differ significantly in 2022 compared to 1998, proximal involvement (57.7% vs. 79.5%) and total occlusion (26.8% vs. 52.7%) were less frequently observed in 2022 (*p* < 0.001) and referral to bypass grafting (CABG) decreased by >50% from 1998 to 2022 (15.5% vs. 39.8% *p* < 0.001). **Conclusions:** Over a 25-year period, the incidence of total coronary artery occlusion and the need for CABG among adults with FH from a high-prevalence founder population were reduced by more than 50% and occurred in older ages. However, the absence of major improvement of coronary anatomy severity underscores the persistently high cardiovascular risk in FH patients.

## 1. Introduction

Familial hypercholesterolemia (FH) is a codominant monogenic disease characterized by lifelong elevated levels of plasma pro-atherogenic low-density lipoprotein cholesterol (LDL-C). When left untreated, heterozygous FH (HeFH) patients will maintain LDL-C levels around 8 to 15 mmol/L, which is associated with a high risk of premature coronary heart disease (CHD), defined as CHD before the age of 55 years in men and 65 in women [1,2,3]. However, the first coronary event can still occur as early as the third decade of life in HeFH [4]. With a global prevalence recently estimated at 1 in 311, FH is the most common autosomal genetic disorder, although its prevalence is significantly higher in certain founder populations, such as the French Canadians, where it is estimated to be around 1 in 120 [5,6].

Elevated LDL-C levels (>5.0 mmol/L) or a diagnosis of FH are well-established markers of more severe CHD [7,8]. However, there are limited data describing the coronary anatomy and severity of CHD in FH, particularly for the pre-modern lipid-lowering therapies era [9]. Over the past 20 years, significant advances in the pharmacological management of FH have been made with the introduction of various treatment avenues, including high-potency statins, ezetimibe and PCSK9 inhibitors, showing remarkable results in HeFH [1,10]. In individuals with FH, the cardiovascular benefits of high-potency statins appear to derive from the stabilization and regression of atherosclerotic plaques [11,12]. Broader trials, which include but are not exclusive to individuals with FH, have demonstrated that PCSK9 inhibitors also lead to plaque volume reduction, as shown by intravascular ultrasonography in statin-treated CHD patients with stable disease [13] and acute myocardial infarction [14]. Considering the implementation of various national and international practice guidelines as well as diagnostic tools developed alongside these pharmacological advances, FH is increasingly being recognized earlier, allowing for more timely and effective management [1,3].

However, the impact of these evolving therapies on the anatomical severity of CHD in FH remains unclear. The primary goal of this study is to determine if coronary lesions in HeFH patients who underwent coronary angiography in the last five years are significantly different from those of HeFH patients who underwent coronary angiography at the same hospital 25 years earlier, within the French-Canadian population, a founder population with high prevalence of FH.

## 2. Methods

### 2.1. Subjects

Subjects included in this cross-sectional study were FH patients who underwent coronary angiography at the Chicoutimi Hospital, the only referral center in tertiary cardiology and clinical lipidology for the Saguenay-Lac-Saint-Jean (SLSJ) region (Quebec, Canada). Those patients were issued from two distinctive cohorts, as previously described [15]. Briefly, the first cohort (1998 cohort) included 240 HeFH patients who had an angiography between 1995 and 1998 [16], whereas the second cohort (2021 cohort) included 108 HeFH patients who consecutively required a coronary angiography between 2017 and 2021. To be included in the current study, subjects must be 18 years of age or older, have a molecularly or a clinically confirmed diagnosis of FH according to FH Canada criteria [17] and have a clinical CHD event (stable angina, unstable angina, non-ST elevated myocardial infarction and ST-elevation myocardial infarction) confirmed by coronary angiography. Subjects with inadequate angiography films for review were excluded. None of the subjects were included in both cohorts. This project (#2022-013) was approved on 18 May 2022 by the Chicoutimi Hospital Ethics Committee in accordance with the Declaration of Helsinki.

### 2.2. Data Collection

Clinical and biochemical data recorded on the coronary angiography day were collected for both cohorts. Data were taken from the existing research database for the 1998 cohort, whereas data from the cardiovascular disease clinic database already used for patients’ follow-up at Chicoutimi Hospital were collected for the 2021 cohort, as previously described [15].

Coronary angiography images were individually reviewed by the research team to obtain anatomical coronary data. Coronary lesions were considered significant if they caused a ≥ 50% stenosis of the vessel. The validated SYNTAX score for coronary disease severity and complexity was attributed to each patient in the 2021 cohort [18,19]. SYNTAX score tertiles are ≤22, 23–32 and ≥33, with increasing risk of major cardiovascular adverse events (MACE). Patients with previous CABG were attributed a SYNTAX score of 23 since this is the usual threshold where bypass surgery is recommended over angioplasty [20]. SYNTAX scores were not available for the 1998 cohort.

### 2.3. Statistical Analysis

Continuous variables were reported as mean ± SD and categorical variables as proportions (%). Group differences for categorial and continuous variables were documented with the Pearson Chi-square statistic and Student’s unpaired two-tailed *t*-test, respectively. In the contemporary cohort, the anatomical severity was analyzed according to (1) the presence of a treatment on admission using a *t*-test with the SYNTAX score and (2) the potency of statin therapy using a one-factor ANOVA with the quantitative SYNTAX score and a Chi-square test with the number of lesions. A *p*-value < 0.05 was used as the significance threshold for every statistical operation. All analyses were performed using SPSS package version 25 (IBM Corp., Armonk, NY, USA).

## 3. Results

Most FH patients hospitalized for CHD were men, with a higher male predominance in the 1998 cohort (76.7% vs. 63.9%; *p* = 0.01). Patients were aged 50.1 ± 11.3 years in 1998 and 60.6 ± 10.6 years in 2021 (*p* < 0.001). The number of patients that fulfilled the criteria for premature CHD dropped from 75% in 1998 to 38% in 2021 (*p* < 0.001). Moreover, the proportion of the even earlier “CHD of the young”, that is CHD < 45 years of age [21], went from 34.6% in 1998 to 10.2% in 2021 (*p* < 0.001). The prevalence of diabetic and obese patients was higher in 2021 (*p* < 0.05) while the proportion of smoking and hypertension was similar. LDL-C concentrations were 6.7 ± 2.1 mmol/L in 1998 and 3.5 ± 1.7 mmol/L in 2021 (*p* < 0.001). The proportion of patients on lipid-lowering therapies at the time of admission was significantly higher in the 2021 cohort compared to the 1998 cohort (66.7% vs. 25.8%, *p* < 0.001). Most treated patients were on statins (Table 1). In the 2021 cohort, patients on statins were predominantly (62.5%) on a high-intensity dose, while only a few (4.7%) were on a low-intensity therapy.

**Figure 1:**
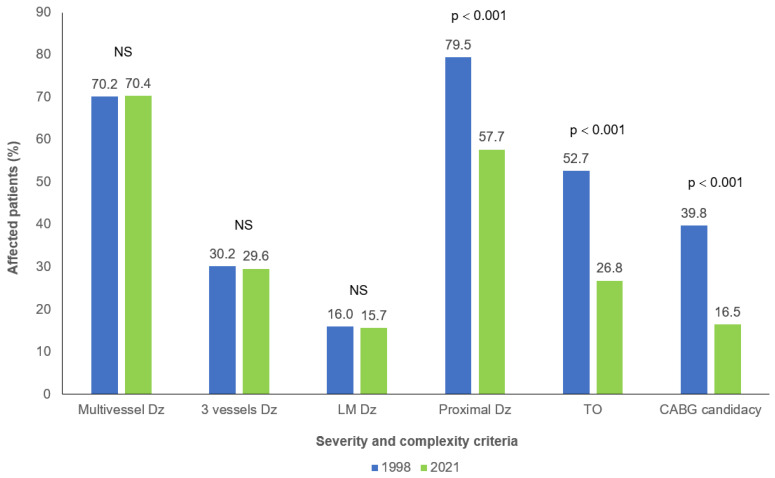
Coronary anatomy severity and complexity characteristics in patients with familial hypercholesterolemia and coronary artery disease between 1998 and 2021. NS: *p* > 0.1. CABG: Coronary artery bypass graft; Dz: Disease; LM: Left main; TO: Total occlusion.

The mean number of coronary lesions (2.5 ± 1.5 in both cohorts) as well as the proportions of multi-vessel (70.2% in 1998 vs. 70.4% in 2021), three-vessel (30.2% in 1998 vs. 29.6% in 2021) and left main involvement (16.0% in 1998 vs. 15.7% in 2021) were not significantly different between 1998 and 2021 (Figure 1). No significant difference was noted between both cohorts when the number of lesions were separated in categories: in 1998 and 2021, respectively, 52.8% and 59.8% had 1 or 2 lesions, 36.4% and 29.9% had 3 or 4 lesions while 10.8% and 10.3% had 5 or more lesions. However, significant decreases in proximal vessel involvement and the presence of total occlusion were observed in patients from the 2021 cohort compared to those in the 1998 cohort (*p* < 0.001) (Figure 1).

As shown in Table 2, the main outcomes and CHD severity were similar between both cohorts regardless of the CHD onset (i.e., premature or late). Interestingly, a trend towards less severe disease was observed in late-onset patients of the 2021 cohort, as indicated by a lower prevalence of multivessel disease and fewer lesions.

**Figure 2:**
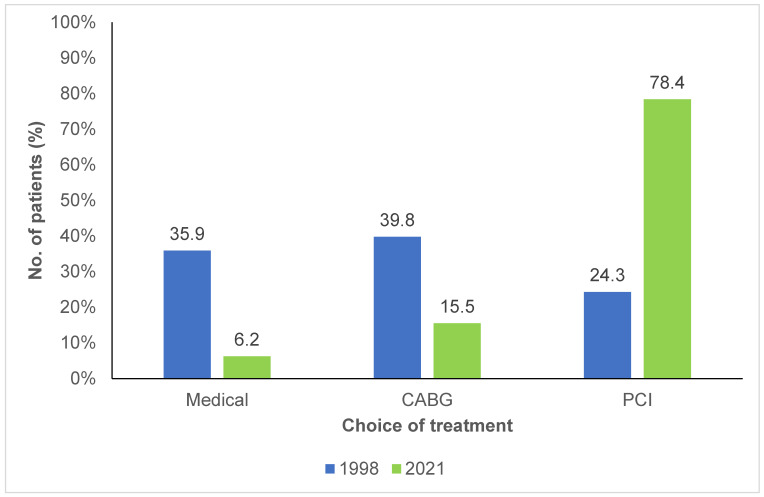
Choice of treatment for patients with familial hypercholesterolemia and coronary artery disease between 1998 and 2021. CABG: Coronary artery bypass graft; PCI: Percutaneous coronary intervention.

As shown in Figure 2, 39.8% of patients in the 1998 cohort were candidates for CABG, compared to 15.5% in the 2021 cohort (*p* < 0.001). In 1998, only a small number of patients underwent percutaneous coronary interventions (PCI), compared to the 2021 cohort, where the proportion had significantly increased, with 78.4% of the patients undergoing PCI.

The presence of lipid-lowering therapy significantly lowered LDL-C in both cohorts (7.28 ± 2.08 mmol/L without treatment, 5.04 ± 1.25 mmol/L with treatment, in 1998; 4.70 ± 1.89 mmol/L without treatment, 2.90 ± 1.31 mmol/L with treatment in 2021; *p* < 0.001 for both). If we further dichotomize for premature vs. late onset CHD, 45% of late onset CHD patients were on lipid-lowering therapy in 1998 and 70.1% in 2021 (*p* = 0.004), while 19.4% of premature CHD patients were on lipid-lowering therapy in 1998 and 61% in 2021 (*p* < 0.001). The number of coronary lesions in both cohorts were similar despite the presence of therapy (*p* > 0.1). Also, in the 2021 cohort, the presence and intensity of statin therapy (low, moderate, high) was not associated with the qualitative number of coronary lesions (*p* = 0.24).

FH patients in the 2021 cohort had a mean SYNTAX score of 14.30 ± 9.18 that indicates a coronary disease of low severity. Patients on high-intensity statins did not have lesser SYNTAX scores than patients on lower intensity or even no statin (12.67 ± 8.99 with no statin, 17.33 ± 6.03 with low, 14.38 ± 8.36 with moderate and 15.81 ± 9.91 with high-intensity statin therapy, *p* = 0.428).

## 4. Discussion

The current study shows that, although the severity of CHD in HeFH patients appears to have remained broadly the same according to conventional criteria, various components of the coronary burden of HeFH patients in a French-Canadian founder population have significantly decreased over 25 years. It was first observed that FH patients hospitalized for a CHD event in 2021 are significantly older by almost 10 years. The mean age for FH patients with CHD events is similar to what was observed in other cohorts [22,23,24]. This increase in the age at hospitalization for CHD results in a remarkable reduction in the prevalence of premature CHD from 75% in 1998 to 37.4% in 2021 and of even earlier-onset “CHD of the young” from 34.6% in 1998 to 10.2% in 2021. Following these findings in the general population [25], diabetes and obesity were more prevalent in the 2021 cohort indicating that FH patients seem to be more prone to metabolic syndrome risk factors. In both cohorts, treatment had a significant effect on LDL-C levels at the time of admission, but modern FH patients had three times more prescription of lipid-lowering treatment, and most patients received high-intensity statins, resulting in twice as lower LDL-C levels. It has been well established from the INTERHEART study that smoking and high cholesterol are the most significant cardiovascular risk factors, accounting for two-thirds of the risk, with a lesser influence for diabetes and obesity [26]. It can therefore be hypothesized that a previous optimized treatment of dyslipidemia in the 2021 cohort may still explain their higher age at presentation despite a higher prevalence of the metabolic syndrome components. However, these findings highlight the ongoing need for better primary prevention of other cardiovascular risk factors in FH patients, given their higher baseline risk. Despite the increased use of statins in the modern cohort, approximately one-third of patients were not receiving any treatment at the time of admission, and the use of advanced therapies, such as PCSK9 inhibitors, remained very low. This might explain the reason behind the persistent levels of LDL-C, which exceeded the recommended targets for FH [1,3,27], indicating an area that requires further improvement.

FH patients have extensive CHD as evidenced by a prevalence of multi-vessel involvement of more than 70% in both cohorts. There was also a high prevalence of three-vessel and left main disease. The proportion of multi-vessel disease observed in cohorts of the current study seems higher than what can be found in other populations. The Danish and French myocardial infarction registries have previously reported a prevalence of multi-vessel involvement in approximately 50% of HeFH patients, despite documenting lower usage of lipid-lowering therapy beforehand as well as reporting similar age and comorbidities in these registries [22,23,24]. Surprisingly, the proportions of multi-vessel, three-vessel and left main disease were almost identical between groups, showing an apparent absence of improvement in gross coronary anatomical severity over 25 years. Additionally, the severity of the disease was comparable between both cohorts regardless of whether CHD onset was premature or late. However, an interesting trend towards milder disease was observed in late onset patients from the 2021 cohort, a pattern not seen in those with premature CHD, despite both groups experiencing a similar increase in prior lipid-lowering therapy. This could be explained by the fact that patients who experience a coronary event at early age might have an inherently more severe disease perhaps due to higher LDL-C concentrations that prevent the observation of significant differences between both cohorts. Despite the absence of gross improvement, proximal vessel involvement and total occlusions were significantly less present in 2021, consequently there seems to be a slight improvement using more refined severity criteria. There was also a dramatic decrease in CABG surgery to treat FH patients in 2021. Since CABG surgery remains the gold standard for multi-vessel, severe and complex CHD [20], CABG candidacy was used as a surrogate marker of coronary severity and its decrease might also show improvement. It is however important to note that medical practice has changed over 20 years and the utilization of CABG has considerably declined in favor of more complex PCI as a means of revascularization since the turn of the millennium [28]. This evolution of practice might explain the decline of CABG candidacy and the rise in PCI in this study.

The SYNTAX score is a surrogate for severity since it serves as a decision-making tool to choose between PCI and CABG, with a low score signifying a favorable chance of MACE with PCI and CABG [18,19], therefore usually representing and anatomy of low severity. The median SYNTAX score obtained in the 2021 cohort sits between what was reported by Farnier [22] and Chieng [7]. There was also a similar presence of bifurcation lesions compared to the French registry, which already showed a significantly higher proportion in FH patients compared to control [23].

The presence of lipid-lowering treatment at the time of admission was associated with favorable lipid profiles in both cohorts but surprisingly did not seem to play a significant role in mitigating CHD severity, so that patients on statins did not have a lower total of coronary lesions nor less multi-vessel or left main disease. This was also true for statin intensity in the 2021 cohort, in which patients on high-intensity statins did not have lesser SYNTAX scores than patients on lower intensity or even no statin. The total duration of lipid-lowering therapy was unknown, and its favorable effect on the lipid profile of on-treatment subjects, especially in the 2021 cohort, was perhaps too recent to have an impact on coronary anatomy. The lack of direct correlation between the improved LDL-C levels and reduced coronary lesion severity could be, in addition to the above reasons, due to multiple factors, such as an established plaque burden or calcified plaques, which could sometimes be resistant to regression despite the lipid-lowering therapies. Also, non-LDL-C mediated factors such as lipoprotein (a), triglycerides, and inflammation, among other lipid parameters, could also play an important role in the disease severity, therefore influencing the reported SYNTAX scores for the targeted population. Overall, despite improved prior treatment, contemporary FH patients from 2021 exhibit CHD that is objectively almost as severe as that observed in patients from 1998, with the only notable improvements being a lower prevalence of proximal disease and total occlusions. These findings still represent an important step forward, mainly when considering PCI, which might be less technically complex. The major improvement shown in this study is, therefore, not on CHD severity itself, but in the older age of patients from the 2021 cohort. While FH patients in both cohorts exhibit the same coronary burden, those in the 2021 cohort experience CHD events approximately 10 years later in life. Higher prevalence of metabolic syndrome risk factors in contemporary patients may also play a role in the similarity of CHD severity, negatively compensating for the effect of improved lipid control. Better holistic primary prevention care should, therefore, be a priority in FH patients. However, considering that the lipid-lowering therapies’ presence and potency were not significantly associated with milder CHD severity suggests an underlying higher cardiovascular risk inherent to FH itself, independent of treatment. The possible legacy effect of the high cumulative LDL-C burden that these patients have from an early age might explain this, as many of them only obtain treatment in their adult years. Once again, this underscores the gap between guidelines and practice, as shown in another recent cross-sectional study [29]. Though the very significant reduction of premature CHD and higher prior use of lipid-lowering therapy in these young patients are encouraging, FH patients must still be screened and identified as early in their life as possible to mitigate their risk. Not to mention the importance of achieving optimal LDL-C targets even in patients presenting a clinical CHD event but with non-obstructed coronary arteries (MINOCA) having less than 50% of reported stenosis. These interesting observations showed that nearly half of MINOCA patients with recurrent acute myocardial infarction (AMI) demonstrated atherosclerosis progression, often requiring revascularization, and only a minority of these cases have achieved the recommended LDL-C targets at the time of recurrent AMI [30].

As mentioned above, HeFH patients still failed to achieve LDL-C targets in 2021 [1,3,27], and the use of PCSK9 inhibitors remained very limited. Therefore, improved access to advanced and emergent lipid-lowering therapies for FH patients should remain a priority.

The current study consists of an important addition to the scarce literature describing the severity and complexity of CHD in HeFH patients [2,22,23,24]. Its main strength lies in its results obtained among a sample of FH patients issued from a well-known founder population with a high prevalence of FH, a homogeneous genetic structure, a relative geographic stability, and a more uniform environmental exposure. The reliability and reproducibility in the interpretation of angiographic data are also important elements of strength for this study. All coronary angiograms of the 2021 cohort were reinterpreted by the same observer from the research team. It was impossible to perform such validation for the 1998 cohort. However, all interpretations were performed by a single person. Furthermore, there is continuity in the method used to interpret a coronary angiogram since the 90s, and the percentage of vessel stenosis is still estimated visually with a significant lesion defined as at least 50% stenosis with a minimum of 2.5 mm in a vessel’s diameter. The SYNTAX score did not yet exist at the time of data collection for the 1998 cohort, so it was impossible to calculate it, depriving us of this reliable and quantitative measure of CHD severity for this cohort. As previously discussed, criteria used for assessing the presence of FH in the 2021 cohort were mostly based on clinical criteria, whereas a molecular diagnosis was available for almost all patients of the 1998 cohort [15]. Some patients also had an earlier CHD event, CABG, or PCI, introducing a bias towards a falsely older age of presentation and more severe anatomy. However, this limitation applies to both the 1998 and the 2021 cohorts, reducing its relative effect on our comparisons. As mentioned earlier, patients with previous CABG were attributed a SYNTAX score of 23 empirically, which might have tilted the mean scores downward if the anatomy was more severe than estimated. The fact that the mean SYNTAX score in the 2021 cohort was close to what was reported in other studies [7,22] indicates the relatively low effect of this bias. Finally, as mentioned above, the criteria for referring a patient to CABG have changed since the 90s [28], and it is an imperfect surrogate marker to compare the severity of coronary anatomy between the two cohorts. It is also worthwhile mentioning that an additional limitation to the study is solely relying on the angiographic assessment of atherosclerotic plaques as the imaging technique used for this study. While angiography remains the gold standard for such clinical purposes, it does provide limited information on plaque morphology and composition. Other advanced imaging techniques, such as intravascular fractional ultrasound (IUVS), fractional flow reserve (FFR), and instantaneous wave-free ratio (iFR) provide high-quality information on plaque vulnerability and burden. The absence of these additional diagnostic tools could represent another explanation for the observed relationship between LDL-C reduction due to LLTs and coronary lesion severity. More efforts should be invested in leveraging these advanced technologies for optimal monitoring of coronary atherosclerotic progression with better appreciation of subtle changes in plaque characteristics.

In summary, various components of the coronary burden of HeFH patients from a French-Canadian founder population have significantly improved over 25 years. The older age of contemporary patients along with reductions in proximal vascular involvement and total occlusions suggest a modest decrease in disease severity, which partially explains the reduced need for bypass surgery. However, the absence of major improvement of coronary anatomy severity despite advancements in treatment underscores the persistently high cardiovascular risk in FH patients. Such observation highlights the need for even earlier and more holistic preventive care, including treating other risk factors as well as improving access to advanced therapies for patients with familial hypercholesterolemia.

## Figures and Tables

**Table 1:** Baseline demographics, comorbidities, lipid profiles and therapies.

Characteristics	1998 Cohort (n = 240)	2021 Cohort (n = 108)	*p*-Value
Men (%)	76.7	63.9	0.01
Age (years, mean ± SD)	50.1 ± 11.3	60.6 ± 10.6	<0.001
Premature CHD (%)	75.0	38.0	<0.001
Age <45 years old (%)	34.6	10.2	<0.001
Diabetes (%)	10.0	17.8	0.04
Smokers (%)	28.2	30.8	NS
Hypertension (%)	39.6	46.7	NS
Obesity (%)	18.8	34.3	0.002
LDL-C (mmol/L, mean ± SD)	6.7 ± 2.1	3.5 ± 1.7	<0.001
LDL-C > 5.0 mmol/L (%)	80.7	22.4	<0.001
Lipid-lowering treatment at admission (%)	25.8	66.7	<0.001
Statin (%)	22.1	59.3	<0.001
PCSK9 inhibitors (%)	0.0	6.5	<0.001
Ezetimibe (%)	0.0	22.2	<0.001
Fibrates (%)	3.8	0.9	NS

NS: *p* ≥ 0.05. Premature coronary heart disease (CHD) refers to CHD <55 years old in men and <65 years old in women. LDL-C: Low-density lipoprotein-cholesterol; SD: standard deviation.

**Table 2:** Severity of coronary heart disease in patients with familial hypercholesterolemia among patients with premature and late onset of the coronary event.

	Premature CHD			Late-Onset CHD		
	1998 (n = 180)	2021 (n = 41)	*p*-Value	1998 (n = 60)	2021 (n = 67)	*p*-Value
Multivessel Dz (%)	66.0	70.7	NS	83.7	70.1	0.093
3 vessels Dz (%)	26.3	24.4	NS	42.9	32.8	NS
LM Dz (%)	13.4	9.8	NS	24.5	19.4	NS
No. of lesions (%)			NS			0.063
1–2	58.4	65.8		34.8	55.9	
3–4	30.9	26.3		54.3	32.2	
≥5	10.7	7.9		10.9	11.9	

NS: *p* ≥ 0.05. Premature coronary heart disease (CHD) refers to CHD <55 years old in men and <65 years old in women. CHD: Coronary heart disease; Dz: Disease; LM: Left main; No: Number.

## Data Availability

The datasets used and/or analyzed during the current study are available from the corresponding author upon reasonable request.
